# Heterogeneity in depression treatment trajectories and associated social factors: a longitudinal cohort study in older adults in Denmark

**DOI:** 10.1192/j.eurpsy.2025.467

**Published:** 2025-08-26

**Authors:** K. Ishtiak-Ahmed, C. Rohde, O. Köhler-Forsberg, K. S. Christensen, C. Gasse

**Affiliations:** 1 Department of Affective Disorders, Aarhus University Hospital Psychiatry; 2 Department of Clinical Medicine, Aarhus University; 3 Psychosis Research Unit, Aarhus University Hospital Psychiatry; 4 Research Unit for General Practice; 5 Department of Public Health, Aarhus University, Aarhus N, Denmark

## Abstract

**Introduction:**

While depression trajectories have been extensively studied in recent decades, research has predominantly focused on younger and middle-aged individuals, often overlooking vulnerable older patients. Classifying patients based on treatment trajectories may enhance personalized care efforts and long-term treatment management for older adults.

**Objectives:**

This study investigates the varying patterns of depression treatment trajectories and examines the influence of social factors on these trajectories in older adults initiating first-time depression treatment over a three-year period.

**Methods:**

We conducted a nationwide cohort study using Danish registers, including all adults aged 65 and older who filled their first-time antidepressant prescriptions between 2006 and 2015 (with no prescriptions in the previous decade). Depression treatment patterns were assessed through antidepressant prescription redemptions and psychiatric hospital contacts for depression. Latent class growth modeling identified distinct treatment trajectories over the three years, while multinomial logistic regression analyzed the association between social factors and trajectory group membership.

**Results:**

Among the 66,540 older adults included in the study (55.2% female, mean age: 77.3 years), three unique depression treatment trajectories emerged: ‘brief treatment’ (33.7%), where treatment ended within six months; ‘gradual withdrawal’ (26.5%), where treatment tapered off over two years; and ‘persistent treatment’ (39.8%), where treatment continued throughout the three years. Association analyses showed that female sex, living alone, and residing in less-urbanized regions were associated with higher odds of membership in the persistent treatment group. In contrast, older individuals, those who were widowed or separated, and individuals of non-Danish ethnicity were associated with lower odds of membership in the persistent treatment group.

**Conclusions:**

This study identifies three distinct depression treatment trajectories in older adults. Social factors such as sex, household composition, place of residence, and ethnicity were associated with treatment duration and trajectories. Tailored interventions based on patient characteristics may enhance depression care for older adults, ensuring more personalized and effective treatment strategies.

**Disclosure of Interest:**

K. Ishtiak-Ahmed: None Declared, C. Rohde Grant / Research support from: CR received the 2020 Lundbeck Foundation Talent Prize, O. Köhler-Forsberg Speakers bureau of: OKF reported honoraria for lectures for Lundbeck Pharma A/S and consultant fees for WCG Clinical, all unrelated to the present work., K. Christensen : None Declared, C. Gasse: None Declared

